# Atypical Small Acinar Proliferation: Repeat Biopsy and Detection of High Grade Prostate Cancer

**DOI:** 10.1155/2015/810159

**Published:** 2015-09-14

**Authors:** Andrew Leone, Katherine Rotker, Christi Butler, Anthony Mega, Jianhong Li, Ali Amin, Stephen F. Schiff, Gyan Pareek, Dragan Golijanin, Joseph F. Renzulli

**Affiliations:** ^1^Division of Urology, Rhode Island Hospital and The Warren Alpert Medical School of Brown University, Providence, RI 02905, USA; ^2^The Warren Alpert Medical School of Brown University, Providence, RI 02905, USA; ^3^Department of Hematology/Oncology, The Miriam Hospital, Providence, RI 02905, USA; ^4^Pathology and Laboratory Medicine, Rhode Island Hospital, Providence, RI 02905, USA; ^5^Section of Minimally Invasive Urology, The Warren Alpert Medical School of Brown University, Providence, RI 02905, USA

## Abstract

*Purpose*. Atypical small acinar proliferation (ASAP) is diagnosed in 1-2% of prostate biopsies. 30–40% of patients with ASAP may be diagnosed with prostate cancer (PCa) on repeat biopsy. Our objective was to examine the association between ASAP and subsequent diagnosis of intermediate/high risk PCa. *Materials and Methods*. Ninety-six patients who underwent prostate biopsy from 2000 to 2013 and were diagnosed with ASAP were identified. Clinicopathologic features were analyzed. Comparison was made between those with subsequent PCa on repeat biopsy and those with benign repeat pathology. *Results*. 56/96 (58%) patients had a repeat biopsy. 22/56 (39%) were subsequently diagnosed with PCa. There was no significant difference in patients' characteristics. Presence of HGPIN on initial biopsy was associated with a benign repeat biopsy (68% versus 23%). 17/22 (77%) had Gleason grade (GG) 3+3 disease and only 5/22 (23%) had GG 3+4 disease. *Conclusions*. 22/56 patients (39%) of patients who underwent a subsequent prostate biopsy following a diagnosis of ASAP were found to have PCa. 77% of these men were diagnosed with GG 3+3 PCa. Only 23% were found to have intermediate risk PCa and no high risk PCa was identified. Immediate repeat prostate biopsy in patients diagnosed with ASAP may be safely delayed. A multi-institutional cohort is being analyzed.

## 1. Introduction

There are over one million prostate biopsies performed annually in the United States [1]. It has become clear that the potential risks, costs, and overdiagnosis of insignificant prostate cancer (PCa) have been underappreciated. Recent literature has demonstrated an increase in the incidence of significant postbiopsy infections and the consequences of overtreatment of indolent disease. Post-prostate biopsy hospitalization rates for infectious complications now approximate 1% [2].

Atypical small acinar proliferation (ASAP) is a diagnosis that occurs in about 1-2% of prostate biopsies [3]. The term ASAP, first defined by Bostwick, represents suspicious glands without adequate histologic atypia for a definitive diagnosis of prostate adenocarcinoma [4]. Previous studies have suggested that 17–70% of patients with ASAP have adenocarcinoma present on subsequent prostate biopsies [5, 6]. Current guidelines recommend immediate repeat biopsy within 3–6 months after the initial diagnosis of ASAP [7, 8]. However, up to 80% of the patients who are found to have adenocarcinoma on repeat biopsy have low risk GG 6 PCa [9]. With the recent trend toward adoption of active surveillance (AS) for low risk PCa defined by NCCN criteria, such patients are followed using serum PSA and digital rectal exam (DRE) every 4–6 months with a repeat biopsy in 1 year. However, there is concern with the potential for undersampling of intermediate risk PCa (GG 3+4). Recent evidence suggests that AS may be an acceptable treatment option in select patients with GG 3+4 PCa. Cooperberg et al. demonstrated that when patients do progress, the progression rate is slow and delayed intervention does not impact overall survival [10]. Also, recent research using MRI/TRUS targeted fusion biopsy has confirmed that patients diagnosed with subsequent PCa all had low risk disease [11]. We sought to explore the natural history of ASAP and the infrequent diagnosis of high risk PCa on repeat biopsy.

## 2. Materials and Methods

After obtaining appropriate institutional review board approval, a retrospective chart review of patients of an academic group practice from January of 2000 to December of 2013 was performed. Prostate biopsies were performed using transrectal ultrasound guided prostate biopsy for elevated PSA and/or abnormal DRE (standard 12-core template) and 96 patients were diagnosed with ASAP. Patient-specific data including age, prostate size on transrectal ultrasound, PSA, interval between repeat biopsies, GG on repeat biopsy, and subsequent treatment modality were recorded. Patients who had undergone previous prostate biopsy or those who had concomitant adenocarcinoma on biopsy or ASAP diagnosis prior to 2000 were excluded. Repeat prostate biopsy was performed with standard 12-core template with increased sampling performed at the previous site of ASAP at the discretion of the urologist. The diagnosis of ASAP was confirmed by a single genitourinary pathologist (AA). The pathologist reviewed all slides to confirm diagnosis of ASAP.

Clinicopathologic features recorded included age at biopsy, prebiopsy PSA, DRE findings, number of cores with ASAP, presence of high grade prostatic intraepithelial neoplasia (HGPIN), presence of cancer on repeat biopsy, Gleason score at repeat biopsy, and GG at radical prostatectomy (RP) for patients treated with RP. The primary outcome was detection of intermediate/high risk PCa, defined as GG ≥7 on repeat biopsy, in accordance with prior studies [12, 13]. Secondary outcome was diagnosis of any grade of PCa on repeat biopsy.

Continuous variables were compared using Student's *t*-test and Wilcoxon rank-sum test, and categorical variables were compared using chi-square and Fisher's Exact test, as appropriate. Separate logistic regression analyses were performed to evaluate the associations of clinicopathologic features with subsequent diagnosis of any grade and high risk cancer. Statistical analyses were performed using SPSS (version 21.0 (IBM, Chicago, IL, USA)). All tests were two-sided with *p* values <0.05 considered statistically significant.

## 3. Results

Overall, 96 patients were diagnosed with ASAP. Median age was 62 years (IQR 56, 67), median PSA at presentation was 6.0 ng/mL (4.0,6.8). 17% of patients had abnormal DRE demonstrating cT2a or cT2b disease. Median follow-up was 2.8 years (1.5,3.6) and no deaths were recorded in the cohort (Table 1).

**Figure 1:**
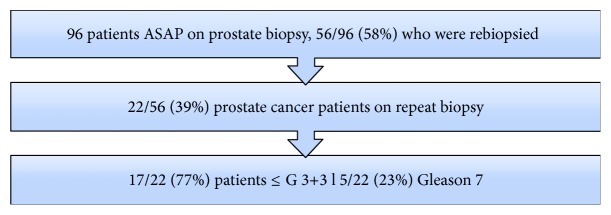
Flowchart of patients diagnosed with ASAP.

Of the 96 patients with ASAP on initial diagnosis, 56 (58%) had a repeat biopsy (Figure 1), with 45 patients undergoing repeat biopsy within 3–6 months of the initial ASAP diagnosis. The reasons for a lack of repeat biopsies were patients' preference and noncompliance. Table 1(b) depicts the comparative demographic data for patients who did undergo repeat biopsy to those who did not undergo repeat biopsy.

Age was statistically different, 62 versus 65 years (*p* = 0.051), but PSA did not differ between the two groups. The remaining 12 patients underwent repeat sampling within 2.5 years. Median time to repeat biopsy was 4.4 months (1.2,5.6). 22/56 (39%) patients were diagnosed with PCa on repeat biopsy. Of those 22 patients, 17 (17%) patients had GG 3+3 disease and 5 had GG 3+4 disease (Table 2). Median number of cores positive was 1.8 (1.0,1.7). None of the cases revealed higher grade prostate adenocarcinoma on repeat sampling. Median PSA prebiopsy was 6 ng/mL (4,6.8).

Radical prostatectomy (RP) was performed in 9 patients, while 3 received external beam radiation. One patient was upgraded from GG 3+3 to 3+4 in radical prostatectomy. No biopsies revealed GG 4+3 or higher in this cohort. The remaining patients underwent active surveillance and remained on active surveillance at last follow-up.

To identify features associated with a subsequent diagnosis of any grade cancer, we examined bivariate associations of clinicopathologic characteristics among men with and without subsequent cancer diagnosis (Table 3). There was no significant statistical difference in the number of initial biopsy cores positive for ASAP between the two cohorts (those with cancer = 1.76 versus those without 1.33, *p* < 0.21). Concomitant HGPIN was identified in 23% of the patients that progressed to PCa compared to 67% of those not diagnosed with cancer on repeat biopsy (*p* < 0.001). Median age and PSA level at time of initial biopsy were not statistically different between the two cohorts.

## 4. Discussion

In this study, we examined the natural history of ASAP and the subsequent diagnosis of intermediate or high risk PCa on repeat biopsy. Notably, only 9% of men who underwent a repeat prostate biopsy were found to have intermediate risk PCa. No cases of high risk PCa were detected. Thus, for every thirteen repeat biopsies, one intermediate risk PCa was detected.

PCa is the leading noncutaneous cancer diagnosis among men and the second leading cause of cancer death in the United States [14]. Recently, the utility of PCa screening has come under scrutiny because of the suspected overdiagnosis and overtreatment of PCa.

ASAP generally encompasses marginally sampled adenocarcinomas or benign acini with reactive atypia or partial atrophy. ASAP differs histologically from PCa since it has a smaller size with fewer acini, more prominent nuclear hyperchromasia, and less prominent nuclear enlargement [15]. Current recommendations for ASAP diagnosis suggest repeating the biopsy within 3–6 months of the initial biopsy. This is based on the study by Iczkowski et al., which revealed that most PCas found in patients with ASAP are detected within the first 6 months after the initial biopsy [16]. Similarly, in the study by Epstein and Herawi, 90% of PCa following an ASAP were found on the first repeat biopsy [17]. This would most likely represent undersampling rather than disease progression. Dorin et al. reported that 51% of men subsequently diagnosed with cancer harbored clinically significant disease by the modified Epstein criteria [18], and Leone et al. noted that 43% of men treated with RP after a subsequent PCa harbored clinically significant disease [19]. Conversely, Warlick et al. observed that only 17.3% of men diagnosed with ASAP were subsequently found to have high grade (Gleason ≥7) cancer [20], and Raskolnikov et al. identified Gleason ≥7 disease in only 5% of patients with ASAP using MRI/TRUS-fusion guided biopsy [21]. While such findings appear to imply disparate conclusions, the differences in interpretation are largely related to choice of denominator. The prevalence of intermediate or high grade PCa is approximately 30–50% among men initially diagnosed with cancer. Also, the prevalence of significant or intermediate or high risk cancer among men with ASAP who undergo repeat biopsy is only 5–20%.

Other studies have suggested that concomitant HGPIN and ASAP increase the risk of PCa in repeat biopsy [22]. Conversely, our study suggests that a finding of concomitant HGPIN confers a statistically significant difference in the identification of PCa in ASAP patients undergoing repeat prostate biopsy (23% versus 67%, resp.) (*p* < 0.001).

Prostate biopsies can be associated with significant morbidity including pain, bleeding, and infectious complications. Also, a recent study has shown appreciable erectile dysfunction after transrectal prostate biopsy as early as three months after the biopsy [23]. Further, with increasing fluoroquinolone resistance and emergence of extended spectrum beta lactamase (ESBL) producing organisms, hospital admission rates following prostate biopsy are increasing and in some countries may be as high as 4% [24]. There are numerous studies demonstrating a relationship between ASAP diagnosis and low frequency of intermediate/high grade PCa [25]. Our study confirms this notion and suggests that, in appropriate patients, a 3–6 month repeat biopsy can be safely delayed.

In our opinion, patients with ASAP can be followed with parameters similar to AS for very low risk or low risk PCa [26]. In our series, the patients who were subsequently diagnosed with PCa after initial ASAP diagnosis were optimal AS candidates given the low volume (low number of cores and percentage of cores) as well as low prebiopsy PSA. We recommend placing newly diagnosed ASAP patients on an AS protocol abrogating the need for immediate repeat biopsy.

The limitations of our study include its retrospective nature and limited number of repeat biopsies. A substantial portion of patients were lost to follow-up. Variability in time to repeat biopsy for ASAP also demonstrates the lack of uniformity in practice patters within a group on academic urologists. There is significant interobserver variability in the diagnosis of ASAP. To address this issue, we utilized one fellowship trained genitourinary pathologist to review all cases and confirm the diagnosis of ASAP prior to their inclusion.

Over the last decade, there has been a trend to more aggressive grading of PCa known as the Will Rogers phenomenon [27]. Further, there has been a paradigm shift in the treatment of low risk PCa in recent years. Current advocacy is for very low and low risk PCa to be treated with AS. Long term follow-up (>10 years) is not available; however, some limited evidence suggests ASAP rarely progresses to intermediate PCa and does not progress to high grade PCa.

Our results demonstrate that a limited number of patients (39%) with an initial diagnosis of ASAP were subsequently diagnosed with PCa. However, only 9% of this patient population was diagnosed with intermediate risk PCa. No high risk PCa was detected. Prospective studies are needed to corroborate the natural history of men diagnosed with ASAP seen in our cohort.

## Figures and Tables

**(a):** 

Parameters	Data (IQR)
Number of patients ASAP on initial biopsy	96
Median number of cores positive ASAP	1.3 (1.0–1.7)
Median time to rebiopsy (months)	4.4 (1.2–5.6)
Percentage of patients repeat biopsy	58%
Median age (yrs.)	62 (56–67)
Median PSA (ng/mL)	6 (4.0–6.8)
DRE abnormal (%)	17%
Median follow-up (years)	2.8 (1.5–3.6)

**(b):** 

	Underwent rebiopsy	No rebiopsy	*p* value
	*N* = 56	*N* = 40	
Age (yrs.) (IQR)	62 (55–67)	65 (57–68)	**0.05**
PSA (ng/mL) (IQR)	6.0 (3.3–6.3)	6 (3.5–6.1)	0.92

**Table 2:** Outcomes of rebiopsy on patients with CaP.

Gleason grade biopsy	Number of patients	RP	Upgraded (RP path)
3+3	17	8	1
3+4	5	1	0

**Table 3:** Comparison of patients with and without PCa and high grade versus low grade PCa on repeat biopsy.

Feature	Any cancer on repeat biopsy		*p* value	GG ≥ 7 on repeat biopsy		*p* value
	(*N* = 56)			(*N* = 5)		
	Yes (*N* = 22)	No (*N* = 34)		Yes (*N* = 5)	No (*N* = 17)	
Mean age (years)	63	60	0.18	65 (59–69)	62 (57–66)	0.58
Abnormal DRE (%)	24	28	0.74	20	23	0.76
Mean PSA (ng/dL)	6	6	—	11 (5–15)	6 (4–7.3)	0.09
Mean # cores with ASAP	1.76	1.33	0.21	1	1.7 (1–3)	0.23
Presence of concomitant HGPIN	**22.7%**	**67.6%**	**0.001**	20%	9%	0.47

## Abbreviations

ASAP: Atypical small acinar proliferation
CaP: Prostate adenocarcinoma
DRE: Digital rectal exam
AS: Active surveillance
GG: Gleason grade
HGPIN: High grade prostatic intraepithelial neoplasia.
